# Using Professional Terms

**Published:** 1899-10

**Authors:** 


USING PROFESSIONAL TERMS.—Not long ago a lady, who had recently had a large filling inserted in one of her bi-cuspid teeth which caused a shock every time she took a drink of cold water, called to ascertain the cause of it. On being informed the cause, she said that she had been back to the dentist who inserted it and he had told her that the tooth "hadn't gotten used to the filling yet."

